# Impact of a movie club on the well-being of Buckinghamshire psychiatry doctors facing moderate-to-high burnout risk

**DOI:** 10.1192/bji.2024.43

**Published:** 2025-08

**Authors:** Sureyya Melike Toparlak

**Affiliations:** Child and Adolescent Mental Health Services, Oxford Health NHS Foundation Trust, Oxford, UK

**Keywords:** Movie, psychiatry, mental illness, well-being, education and training

## Abstract

Burnout is a common issue among healthcare professionals and can have a negative impact on both personal and professional well-being. This initiative follows a group of doctors working in Buckinghamshire, UK, who are at moderate to high risk of burnout, over 6 months to determine whether participation in a movie club, as a form of stress relief and social support, can have a positive impact on well-being. The aim of the project was to investigate the impact on doctors’ well-being by improving connectedness, reducing the feeling of isolation and encouraging face-to-face activities.

Responding to the General Medical Council's National Training Survey 2023, which found that two-thirds (66%) of trainees and over half (52%) of trainers are at high or moderate risk of burnout,^1^ we decided to introduce a movie club. Before this project started, we used the same seven questions from the Copenhagen Burnout Inventory as were used in the annual GMC survey, to record baseline burnout levels of all attendees. Results showed that 55.4% of attendees were at moderate or high risk of burnout, which suggests that the sample was representative of doctors who participated in the national survey. The movie club was conceived as an initiative to address these alarming statistics.

Psychiatry and cinematography are inseparably linked not only because they creatively complement each other, but also as mutual influences blending into didactical categories and professional driving forces, benefiting both the filmmakers’ and psychiatrists’ professions.^2^ Thoughtful viewing of contemporary films and serious discussion of them is a useful and enjoyable teaching format that helps to bridge the gap between the art and science of psychiatry.^3^ A study suggested that movies can be used successfully in teaching psychiatry trainees.^4^ For instance, Psychiatric Hospital Vrapče in Croatia introduced movies successfully in the education of psychiatry residents.^5^ This project was created because of a lack of face-to-face events for colleagues in the post-pandemic era. Apart from its educational benefits, the aim of the study was to investigate its effect on doctors’ well-being.^6^

## The movie club in the making

The Movie Club was designed as a response to the post-pandemic era's lack of face-to-face events for colleagues. This project was well-supported by the medical staffing team, medical directors and college tutors.

Members of the movie club created a list of suitable movies collaboratively. Some of them were *Pity* by Babis Makridis; *Her* by Spike Jonze; *Split* by M. Night Shyamalan; *The Night Listener* by Patrick Stettner; *Girl, Interrupted* by James Mangold and *One Flew Over the Cuckoo's Nest* by Miloš Forman. Moreover, some members added short notes and trailers to help members before they voted for the next event. Movies and event dates were democratically chosen by members via free online poll tools. Posters for each event were created by using an application by the author, printed out in the Whiteleaf Centre library in A3 size and displayed in the doctors’ mess, shared on social media and by word of mouth.

### Consent and ethics

Given that this was an educational project with minimal to no risk, no consent was needed for this project. Participants were informed that they cannot be identified via the manuscript, and that the participants have been fully anonymised by the author. This project aimed to improve the well-being of doctors at moderate to high risk of burnout. The focus was to create a supportive environment rather than conducting formal research that involves patient data or direct clinical interventions. Therefore, institutional review board approval was not required.

## Organisation and participation

The project was well supported, with funding allocated following a proposal submission. Budget was used for technological equipment for the movie club, including, but not limited to, a sound bar, blu-ray player, DVDs, cables, and food and drinks for each session. The meetings took place in one of the consultation rooms in the Whiteleaf Centre in Aylesbury, which was booked via reception for monthly meetings to start at 17.30 h.

Seven participants attended the first movie viewing for *Pity*. Three attended to watch *Donnie Darko*, *A Dangerous Method* and *Mad to Be Normal*, and two attended the last session to watch the movie *Awakenings*.

## Reflections and feedback

Participants consented their information to be used anonymously for this article. Qualitative feedback to the question ‘What are the three things you got out of this session?’ highlighted attendees’ appreciation for a face-to-face interaction with colleagues. One of the recurring topics was enjoying the food and socialising. Some of the reflective feedback were as follows: ‘Chatted with the colleagues in a more relaxed/informal atmosphere’, ‘I felt well rested and relaxed after the movie’ and ‘Good discussion about work and life in general’.

Verbal discussions were held in person following movie viewing. Participants had the opportunity to improve their knowledge not only on curriculum topics such as psychodynamic psychotherapy, psychotic phenomena, the biopsychosocial model, discovery of L-dopa, the anti-psychiatry movement, psychedelics and catatonia, but also on clinically relevant practical areas like boundary crossing, self-disclosure and workplace dynamics. Furthermore, it was a great opportunity to learn more about metaphysics and morphic resonance from knowledgeable colleagues following the viewing of *Donnie Darko*, and explore the intersection between other disciplines and psychiatry.

Regarding quantitative feedback, only data for two participants who attended to three or more sessions were analysed. The change in burnout scores of those participants were negligible.

A follow-up survey was conducted online via link created by an online tool a month after the last event, to assess the effects of this initiative on the attendees. Eight out of 11 participants engaged with the follow-up survey. Survey results suggest the movie club had positive effects on attendees’ well-being, especially on relaxation, connectedness, relationships with colleagues and professional growth. One of the most striking results was that all of the attendees reported that they found the opportunity to network with colleagues valuable during these events. Most of the members reported that the face-to-face social environment positively affected their overall well-being. Attendees also felt more relaxed after the events and found the movie club beneficial for their professional growth. In addition, they felt connected with like-minded individuals and felt more comfortable being around work colleagues through these gatherings.

## Challenges and insights

Although the movie club was advertised via email, posters, social media and word of mouth, attendance was low, with a declining trend from seven participants in the first session to two in the last session. Therefore, its attractiveness and ease of access should be questioned. Potential factors for low attendance rate might include geographical distribution of trainees, distance to event location, on-call schedule and personal commitments. A hybrid option was offered to all members of the group in each session, but demand was limited to only one member. Conducting a survey or verbal interview to explore reasons behind this issue would be beneficial. It is fair to mention that low attendance affected the applicability of this initiative to a wider group. This project was conducted for a relatively short period of time (6 months) because of limitations related to the author's (project lead) rotation duration. Therefore, long-term effects of the movie club could not be explored. Future research should include more extensive data collection, larger samples and longer duration.

In conclusion, social interactions help to reduce stress and promote a healthy work–life balance. They also help doctors to decompress and improve their well-being. Investment in doctors’ well-being can lead to better patient care, more job satisfaction and less severe burnout symptoms, benefitting both doctors and everyone in the healthcare system. This movie club suggested that there may be some benefits of informal face-to-face events on doctors’ well-being. Lack of connectedness with colleagues and continuous pressure at work might lead to burnout in the long term. Therefore, investing in initiatives such as this could be an effective way to strengthen connection between colleagues, create time to unwind and prevent and combat burnout in the healthcare system.
